# A Silicon Resonant Accelerometer Embedded in An Isolation Frame with Stress Relief Anchor

**DOI:** 10.3390/mi10090571

**Published:** 2019-08-29

**Authors:** Jian Cui, Haibing Yang, Dong Li, Ziyang Song, Qiancheng Zhao

**Affiliations:** 1 National Key Laboratory of Science and Technology on Micro/Nano Fabrication, Institute of Microelectronics, Peking University, Beijing 100871, China; 2 Chinese Society of Micro-Nano Technology (CSMNT), Beijing 100084, China

**Keywords:** silicon resonant accelerometer, temperature-induced stress, stress insensitive anchor, isolation frame

## Abstract

Bias thermal sensitivity is a significant performance parameter of a silicon resonant accelerometer (SRA) and is normally used to evaluate the degree of engineering practicability. Theoretical analysis demonstrates that temperature-induced stress is the dominant factor that determines the bias temperature drift of the custom-designed SRA. To solve this issue, this paper presents an SRA embedded in an isolation frame with stress insensitive anchor that prevents the resonant beams suffering from the thermal stress along the sense axis and thus improving the bias stability. Moreover, a high sensitivity device is achieved by integrating the vibrating beams with the comb fingers without conventional additional mass design. The experimental results show that the nominal resonant frequency of the SRA is around 93 kHz with the sensitivity and nonlinearity of 223.7 Hz/g and 5.1‰. The thermal sensitivities of the two resonant beams are −27.6 ppm/°C and −28.8 ppm/°C, respectively, which can be considered as the results owing to temperature change of the Young’s modulus without the thermal stress effect. The bias thermal sensitivity and the stability (1σ) after compensation are tested to be approximately 0.7 mg/°C and 1 mg over the temperature range from −40 °C to 60 °C with ±80 g measurement range.

## 1. Introduction

Silicon resonant accelerometer (SRA) belongs to the generic category of vibrating beam accelerometers (VBA), which so far have demonstrated the right capability of meeting very demanding strategic-grade performance requirements in terms of accuracy, stability, and linearity following the quartz pendulous accelerometer technology [1,2]. VBA devices sense external acceleration by measuring the change in the resonant frequency of beam oscillators under the inertial loading of a proof mass. Compared with the widely used quartz resonant accelerometers, SRA offers more attractive advantages of lower cost, a more miniature size, and their capability of being integrated with the complementary metal-oxide-semiconductor (CMOS) circuit, etc. However, the SRAs appeared to be more sensitive to mechanical environments, especially to the temperature-induced stress caused by the mismatch of the coefficients of thermal expansion (CTE) between the silicon structure and the glass substrate [3,4], thus resulting in the drift of the resonant frequency and deterioration of the temperature performance. 

Considerable research efforts have been devoted to alleviating the effect of thermal stress and reduce the thermal frequency drift of SRA from the aspects of materials, control circuit, packaging, and post-compensation. Composite materials were used in the resonator design to counterbalance the temperature coefficient of silicon [5,6]. Since the resonant frequency of a micro-resonator can be adjusted by electrostatic tuning, the temperature sensitivity of frequency can be compensated by controlling the polarization voltage changing with temperature [7]. A separate isolation structure or platform was utilized to prevent the thermal stress generated during the die attaching process from transferring to the vibration beams of an accelerometer [8,9,10]. Another creative way is to employ a micro-oven with high precision feedback control of temperature to compensate for the resonator frequency drift over a wide temperature range [11]. To further improve the temperature performance of SRAs, some post-compensation methods took advantage of the resonant frequency of an on-chip resonator or the device quality factor as the thermometer to calibrate the bias output, which can avoid the thermal lag between the accelerometer and the external temperature sensors [12,13].

Differing from the previous literatures, in this paper, we focus on the anchor structure design to isolate the vibration beams from the thermal stress along the sense axis, thereby decreasing the frequency drift of the resonators. It is a more efficient and cost-effective means which requires no additional fabrication steps, complex control circuits, or extra isolation structures. Besides, a high sensitivity device is obtained by integrating the vibrating beams with the comb fingers without conventional additional mass design. The paper is organized as follows. Section 2 describes the mechanical structure design and the fabrication of the developed SRA. Detailed experimental results are given in Section 3. The conclusions are made in Section 4.

## 2. Device Design and Fabrication

### 2.1. Mechanical Design

**Figure 1:**
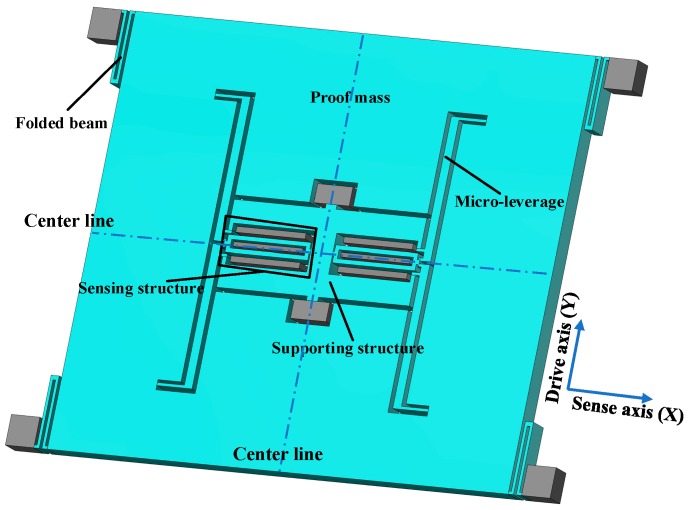
Schematic view of the designed silicon resonant accelerometer.

Figure 1 shows the proposed structure of SRA that mainly consists of a pair of double-ended tuning forks (DETF), a proof mass, two micro-leverages, four folded beams, and a supporting structure. Two identical DETFs are symmetrically distributed on both sides of the geometric center line of the structure. An axial force is generated and coupled onto the resonators when the external acceleration is applied to a movable seismic mass along the sense axis. The resonant frequency of each DETF changes under the applied load. This frequency change is proportional to the input acceleration and serves as the output of the SRA. It should be noted that the two DETFs are arranged collinearly in the sense axis, so they are loaded differentially by the proof mass. That is to say, one DETF is subject to a tension, the other to a compression. This differential configuration doubles the mechanical sensitivity of the SRA and leads a cancellation of common error sources, such as thermal drift and noise to both DETFs.

**Figure 2:**
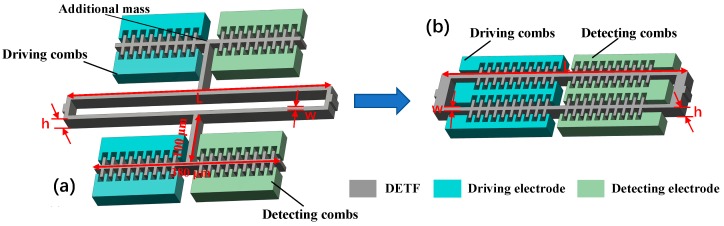
Structure design of DETF. (**a**) Conventional design with airfoil additional mass; (**b**) Improved design with no additional mass.

Silicon-based DETFs are actuated by electrostatic comb finger structures which normally appear as airfoil types with additional mass in the conventional design, as shown in Figure 2a. The comb fingers have both inner and outer stator combs that are fixed to the glass substrate. There are several configurations for the driving and sensing combs. In Figure 2a, the two left groups of comb fingers are used as the driving combs, while the right two groups are the sensing combs. The resonant frequency of DETF is expressed as [14]

(1) $$\begin{array}{l} f=f_{n}\sqrt{1+F\frac{0.293L^{2}}{Ehw^{3}}}\\ f_{n}=\frac{6.96}{\pi }\sqrt{\frac{Eh}{12}{\left(\frac{w}{L}\right)}^{3}\frac{1}{0.398m+m_{a}}} \end{array}$$

where *f_n_* is the natural resonant frequency of DETF without any inertial loading, *E* is the Young’s modulus, *h*, *w*, and *L* are the thickness, width, and length of the vibration beam, respectively. *m* and *m_a_* are the mass of vibration beam and airfoil type combs. *F* is the axial force along the length of the beam and can be written as

(2) $$F=m_{p}a_{ex}A_{F}$$

*m_p_* is the proof mass, *a_ex_* is the external input acceleration, *A_F_* is the mechanical amplified factor caused by a micro-leverage which can further enhance the sensitivity of the accelerometer.

The bias of the SRA is the differential output of the two DFTFs, as follows

(3) $$\Delta f=f_{n}(\sqrt{1+F\frac{0.293L^{2}}{Ehw^{3}}}-\sqrt{1-F\frac{0.293L^{2}}{Ehw^{3}}})$$

Taylor expansion is applied to Equation (3) with the high-order terms omitted, so we obtained

(4) $$\Delta f=f_{n}F\frac{0.293L^{2}}{Ehw^{3}}+\frac{1}{8}f_{n}(F\frac{0.293L^{2}}{Ehw^{3}}{)}^{3}$$

Combining with Equation (2), the scale factor can be written as a ratio of the nominal differential frequency shift between the two resonant force sensors to the applied acceleration

(5) $$SF=\frac{\Delta f}{a_{ex}}\approx 0.187m_{p}A_{F}\sqrt{\frac{L}{Ehw^{3}}\frac{1}{0.398m+m_{a}}}$$

Equation (5) indicates that the scale factor of SRA is determined by the mechanical parameters, but not by any electrical parameter of the circuit. The scale factor and resonant frequency of the vibration beam can be increased by reducing the mass of the airfoil type combs. Hence, an improved structure design is proposed, as shown in Figure 2b, that integrates comb fingers sensing structure into the vibrating beam. The combination of vibrating beam and sensing combs enables additional mass normally used in conventional design not being required, which enhances the sensitivity and ability of anti-vibration by increasing the resonant frequency.

**Figure 3:**
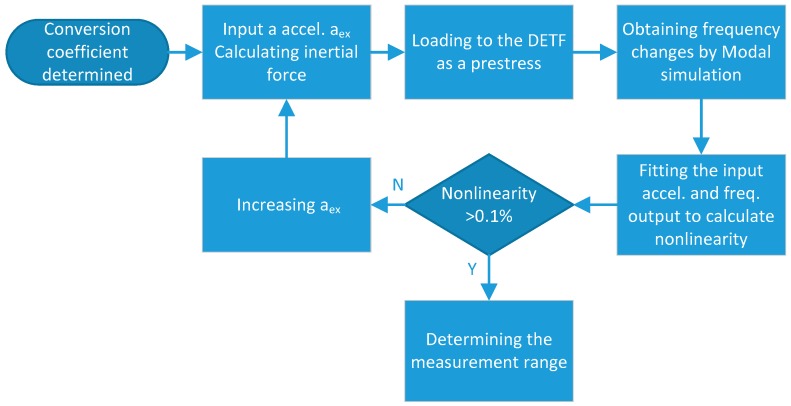
Simulation flow of measurement range.

The measurement range of the SRA is evaluated via a simulation method, as shown in Figure 3. First, the conversion coefficient from the external acceleration input to inertial force applied to the DETF is calculated on the basis of the measured scale factor. Then, a series of inertial forces corresponding to different acceleration inputs can be calculated and exerted on the DETF as a prestressing force, respectively. The frequency variations of the DETF are obtained through modal analysis under distinct prestress force by COMSOL multiphysics (Burlington, MA, USA). The nonlinearity of the frequency variations, with respect to the acceleration inputs, can be evaluated with the simulation results. We selected the nonlinearity of 0.1% as the criterion to define the measurement range in the simulation. When the input is added to 80 g, the simulated nonlinearity increased to about 1.017‰. Thus, ±80 g is considered as the measurement range.

### 2.2. Modal Analysis

The modal analysis of the designed DETF was performed using COMSOL multiphysics. The main structure parameters of improved design with no additional mass (Figure 2b) are listed in Table 1. The dimensions of conventional design with airfoil additional mass are annotated in Figure 2a with the same parameters of *h*, *w*, *L* as the improved design.

**Figure 4:**
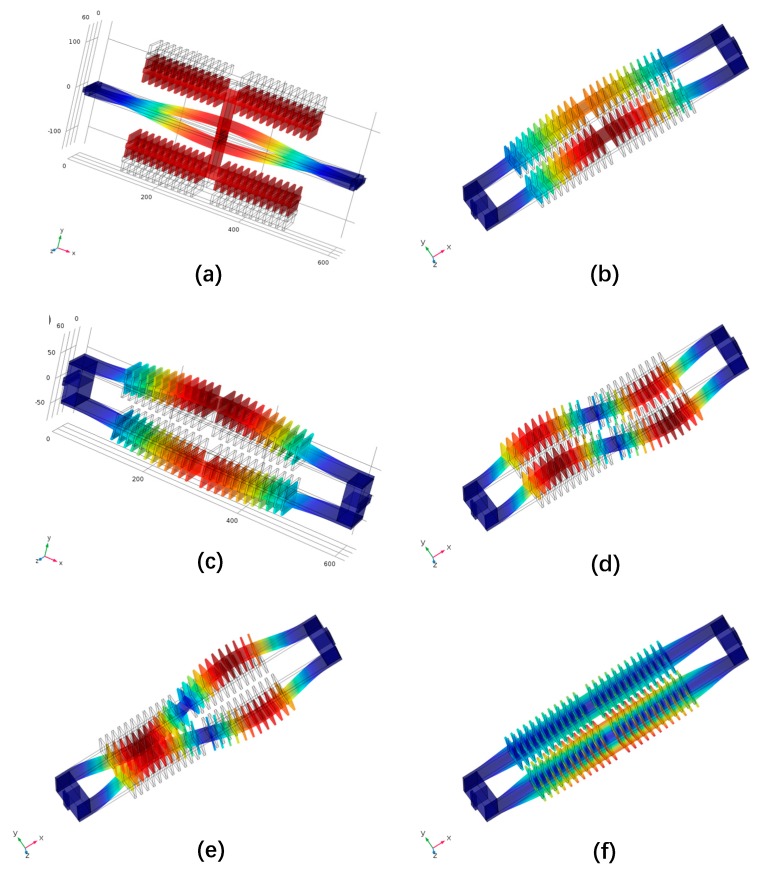
Simulated resonant modes of DETF under no loading force. (**a**) Drive mode of conventional design with airfoil additional mass DETF sensing structure; (**b**) 1st mode: in-plane translation mode; (**c**) 2nd mode: in plane drive mode; (**d**) 3rd mode: out-of-plane in-phase wriggle mode; (**e**) 4th mode: in-plane opposite wriggle mode; (**f**) 5th mode: in-phase torsional mode.

As shown in Figure 4a,c, the working modal frequencies of the DETF without and with additional mass under no external acceleration are 45147 Hz and 93065 Hz. Although both schemes share the identical resonant beam structure parameters (*h*, *w*, *L*), the resonant frequency is doubled by removing the additional mass on DETFs, which can enlarge the scale factor of the SRA and make the device less affected by environment acoustic noise. The first five orders of modal shape of the designed DETF are illustrated in Figure 4b–f, with the simulation results listed in Table 2. The differential actuation configuration of the DETF guarantees that the translation mode cannot be excited. A large frequency split between the high order modes and the drive mode can effectively reduce the interference of other modes to the working mode.

### 2.3. Thermal Sensitivity of Resonant Frequency

On the basis of Equation (1), the temperature sensitivity of the resonant frequency without any axis stress is expressed as the derivative of the frequency, with respect to the temperature, as follows.

(6) $$\frac{1}{f}\frac{\partial f}{\partial T}=\frac{1}{2}\frac{1}{E}\frac{\partial E}{\partial T}+\frac{1}{2}\frac{1}{h}\frac{\partial h}{\partial T}+\frac{3}{2}\frac{1}{w}\frac{\partial w}{\partial T}-\frac{3}{2}\frac{1}{L}\frac{\partial L}{\partial T}$$

As a matter of fact, the thermal sensitivity of the geometric dimension (*h*, *w*, *L*) is equal to the linear thermal expansion coefficient of silicon material α_si_, which is approximately 2.6 ppm/°C [15]. Thus, Equation (6) can be simplified as

(7) $$\frac{1}{f}\frac{\partial f}{\partial T}=\frac{1}{2}\frac{1}{E}\frac{\partial E}{\partial T}+\frac{1}{2}\alpha_{si}$$

It can be shown in Equation (7) that the temperature coefficient of the frequency (TCF) depends on the temperature variation of Young’s modulus of about −60 ppm/°C [16] and coefficient of thermal expansion of silicon material. Therefore, TCF is calculated to be around −28 ppm/°C, which can be used to evaluate the stress of the SRA, since the thermal sensitivity of the bonding stress is much larger than that of Young’s modulus [3]. That is, severe stress may exist in the structure of DETF if the measured TCF of DETF far exceeds −28 ppm/°C, or if there are obvious nonlinear characteristics of the frequency–temperature varying curve.

### 2.4. Stress Insensitive Anchor Design

The anchors are the supporting structures that act as a bridge connecting the sensing elements and the substrate. The anchors offer a unique path of transmitting the temperature-induced stress from the glass substrate to the DETFs. Therefore, the stress can be restrained by the proper design of the anchors.

**Figure 5:**
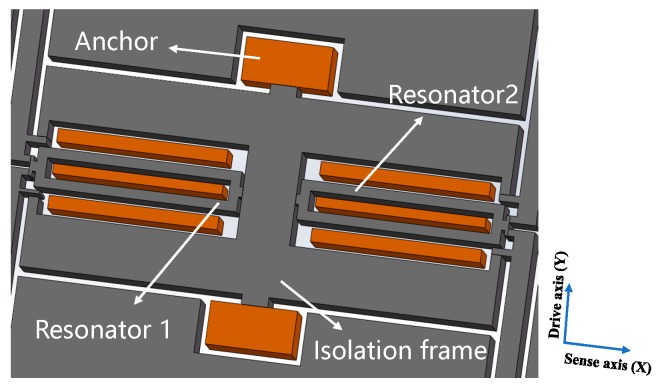
Stress relief anchor design.

The stress relief anchors of the SRA are shown in Figure 5. The internal support structure is implemented with an isolation frame structure, which is located inside the proof mass and outside the DETF resonators. The isolation frame is constructed in the shape of a Chinese character “中” and lies in the center of the accelerometer structure, with geometric symmetry along the drive and sense axis. The entire mechanical structure is suspended above the glass substrate through the anchors that can expand along the drive axis and sense axis, in the case of the thermal stress produced by the mismatch of the thermal expansion coefficients of silicon and glass. Because the stress relief anchors are symmetrically distributed on the isolation frame, of which the stiffness is extremely large, the isolation frame will be deformed by the thermal stress only in the drive axis direction, but not in the sense axis direction. Consequently, the anchors with isolation frame minimize the effect of thermal stress transmitted to the DETF resonators, resulting in reducing the sensitivity of the resonators to the thermal stress.

**Figure 6:**
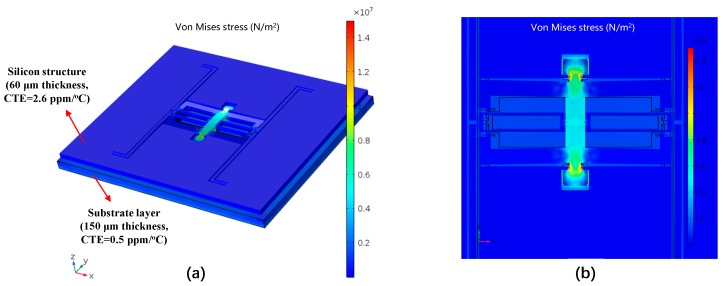
Simulated Von mises stress nephogram of the SRA. (**a**) The thermal stress distribution when the ambient temperature rises from the room temperature to +50 °C; (**b**) Detailed view of the stress distribution on the anchors and isolation frame.

To investigate the effect of the stress relief anchors, the Von mises stress nephogram is obtained by a simulation with COMSOL multiphysics with the ambient temperature rising from the room temperature to +50 °C, as displayed in Figure 6. A small area in the center of the substrate is clamped as the boundary condition. A thick substrate layer is normally adopted to reduce the die stress. In practice, Pyrex 7740 glass (Corning, NY, USA) with a thickness of 500 μm was selected as the substrate, due to its similar thermal expansion coefficient (3.25 ppm/°C) with that of silicon material (2.6 ppm/°C). We intentionally selected a set of worse simulation parameters. The thickness and CTE of the substrate are set as 150 µm and 0.5 ppm/°C, which are less than the practical used value. If the simulation results obtained by this group of worse parameters show a good stress relief effect, the actual stress situation will be better. It can be revealed that, thanks to the utilization of the designed anchors with isolation frame, the temperature-induced stress mainly concentrates on the connection between the anchors and the frame along the drive-axis (Y). The DETF resonators are almost unaffected, which can improve the temperature stability of the SRA.

### 2.5. Fabrication

**Figure 7:**
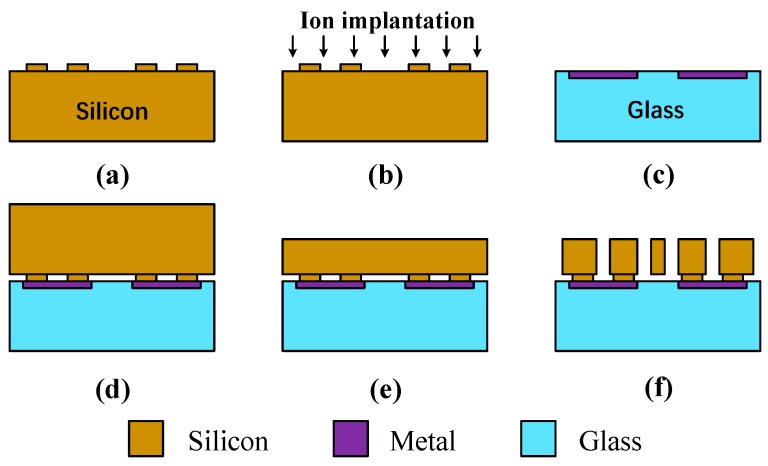
The standard SOG process. (**a**) Etch bonding area on silicon wafer. (**b**) Ion implantation on bonding surface. (**c**) Depositing the metal electrodes on glass substrate. (**d**) Bonding glass substrate to silicon wafer. (**e**) Thinning and polishing silicon wafer. (**f**) Release the structure through DRIE.

The SRA is fabricated with the silicon on glass (SOG) process which is illustrated in Figure 7. The process starts with a high doping and low resistivity silicon wafer. Photoresist defines the anchor area of wafer and deep reactive ion etching (DRIE) process etches the convex plate as the bonding interface (Figure 7a). Ion implantation on the bonding surface could reduce the resistivity at the bonding interface and ensure good ohmic contact (Figure 7b). In order to ensure thermal expansion matching with silicon materials, Pyrex 7740 glass is selected, due to its similar thermal expansion coefficient with silicon material. Graphic transfer of metal electrode on glass is accomplished by the lift-off technique (Figure 7c). After the metal electrode is formed, the silicon wafer and the glass substrate are aligned and bonded, which realizes the electrical connection and electrode extraction between mechanical structure and glass substrate (Figure 7d). 

**Figure 8:**
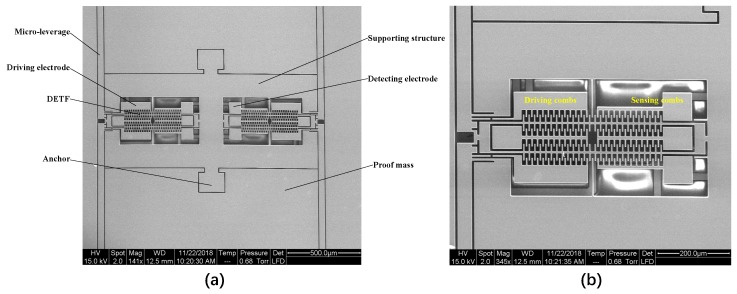
The SEM image of SRA structure. (**a**) Two DETFs embedded in the isolation frame; (**b**) Close ups of the DETF (Left).

Then, the KOH solution is used to reduce the thickness of the silicon structural layer to 60 μm (Figure 7e) and to finally release the mechanical structure through the DRIE process (Figure 7f). The scanning electron microscope (SEM) images of the custom-designed SRA are depicted in Figure 8.

## 3. Results and Discussion

### 3.1. Packaging and Control Circuit

**Figure 9:**
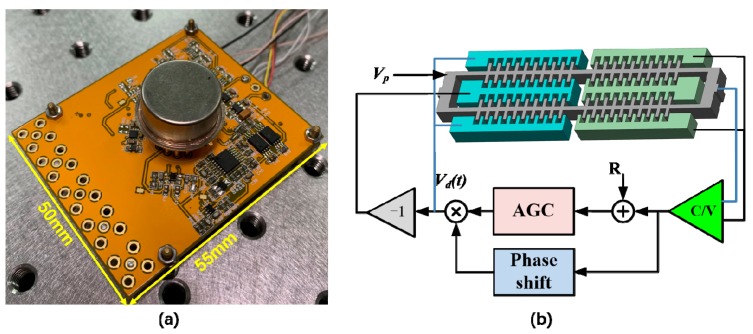
The test circuit for vacuum sealed SRA. (**a**) Test PCB mounted with the SRA; (**b**) Control scheme of self-oscillation loop.

The fabricated SRA is vacuum sealed in a cylindrical metal packaging with the quality factor around 64000 which reduces the frequency noise in the output from phase jitter [1]. A printed circuit board (PCB) is designed to test the performance of SRA with pre-readout circuits and self-oscillation with automatic gain control (AGC) circuit [17], as shown in Figure 9a. The overall size of the test circuit is 55 mm × 50 mm. Figure 9b describes the control scheme of self-oscillation loop. A dc voltage *V_p_* is applied to the proof mass. A continuous-time charge-sensitive amplifier (CSA) is adopted to convert the capacitance variation to voltage (C/V). The displacement voltage is then fed into a 90° phase shift circuit to meet the phase requirement of the Barkhausen criterion. An AGC is used to control the amplitude of the displacement voltage to a reference R by adjusting the amplitude of the drive voltage *V_d_*(t) that is differentially applied back to the driving combs of the DETF.

### 3.2. Test Results

**Figure 10:**
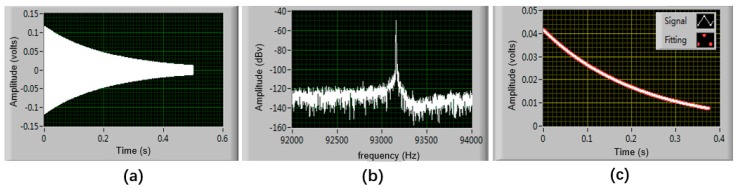
The frequency and quality factor of the DETF1 (Left) testing. (**a**) Ring-down waveform of the SRA vibration; (**b**) Resonant frequency test using FFT; (**c**) Envelope of the attenuation response curve.

A ring-down method has been employed to extract the resonant frequency and quality factor of the SRA, using a NI 6366 data acquisition card [18]. First, an actuation signal with a frequency of around 90 kHz was generated to apply to the drive electrodes of DETF for several seconds, and then it was removed. The frequency of the ring-down waveform (Figure 10a) was determined through the fast fourier transform (FFT) using LabVIEW (National Instruments, Austin, TX, USA) (Figure 10b). The envelope of the attenuation response curve is fitted to calculate Q, as shown in Figure 10c. The resonant frequencies and quality factors of the two DETF resonators without any input acceleration are measured to be 93228 Hz, 64073, and 92847 Hz, 62340, respectively. The measured resonant frequencies are close to the simulation results. The initial frequency split is then calculated to be 381 Hz due to the different fabrication tolerance of the two DETF resonators.

**Figure 11:**
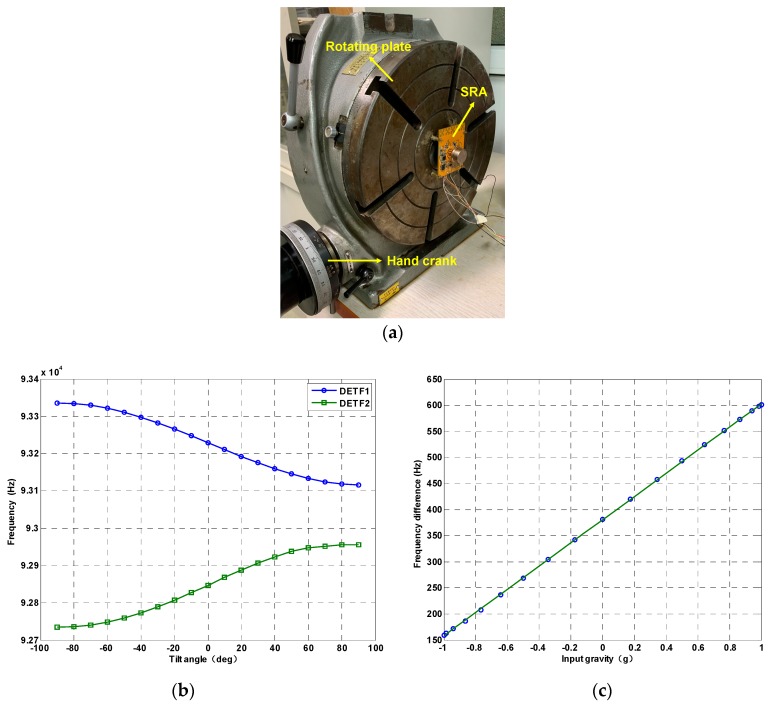
The scale factor testing. (**a**) The SRA is fixed on the indexing plate to test the scale factor; (**b**) The frequency output of two DETFs; (**c**) Frequency difference vs. input gravity.

A NI 6366 data acquisition card (National Instruments, Austin, TX, USA) was adopted to obtain the resonant frequencies of the two DETFS. The test PCB mounted with SRA is installed on an indexing plate which can be precisely changed the sense axis angle from −90 degree to +90 degree with 10-degree intervals by a hand crank, as shown in Figure 11a, thus resulting in 19 test points. At each tilt angle position, the output frequencies of the oscillator are recorded by the frequency counter for 30 s and the mean value of the 30 data is considered to be the final output under a specific position. Figure 11b shows the resonant frequencies variations of DETF 1 and DETF 2 at all tilt angles. Figure 11c demonstrates that the scale factor of the SRA is 223.7 Hz/g with a nonlinearity of 5.1‰.

**Figure 12:**
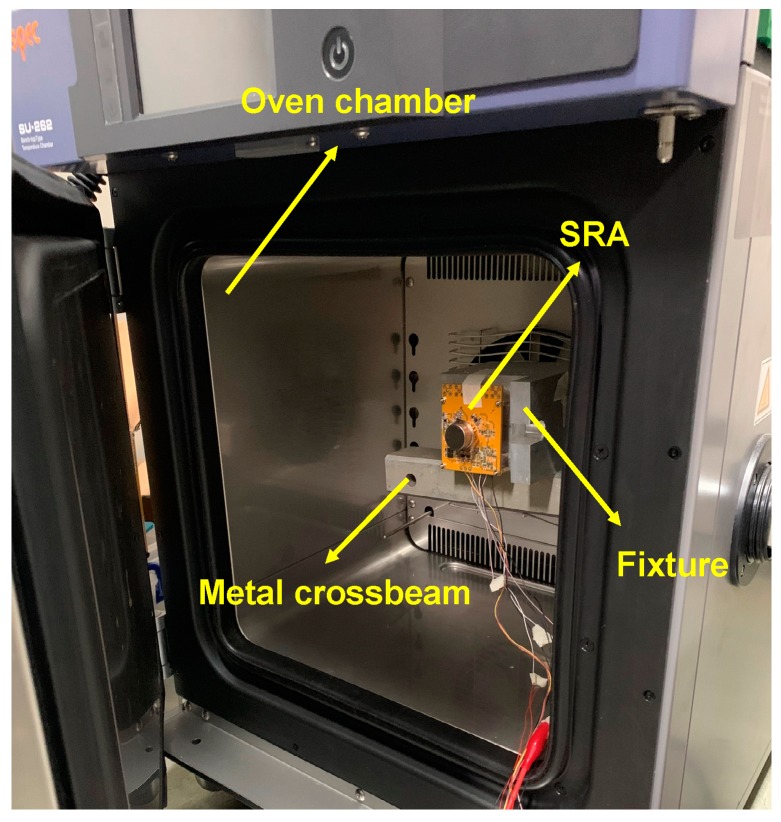
The SRA is put inside an oven chamber for temperature performance testing.

The SRA with the control circuit was put inside an oven chamber to measure the bias temperature performance, as shown in Figure 12. The device is installed on a custom-designed fixture. To keep the SRA away from the vibration of the oven chamber due to its compressor engine operation, the fixture should not be directly contact with the chamber but should be mounted on a metal crossbeam that is isolated from the chamber. The temperature was configured from −40 °C to 60 °C, with the variation rate of 1 °C/min.

**Figure 13:**
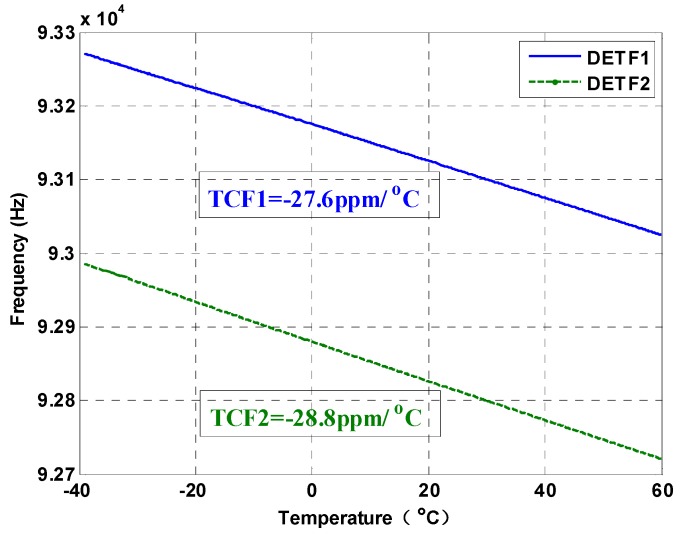
The resonant frequencies of the two DETFs vary with the temperature.

**Figure 14:**
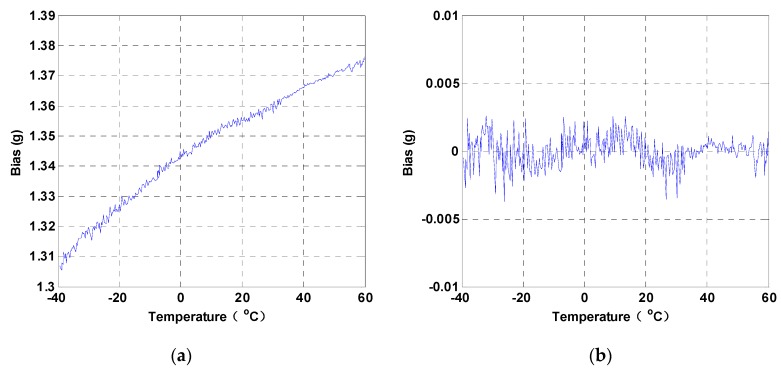
The bias of the SRA vary with the temperature. (**a**) Raw bias output. (**b**) Compensated bias output.

The resonant frequencies of the two DETFs and the temperature information are continuously measured and stored with a sampling frequency of 1 Hz. The varying curves of the resonant frequencies, with respect to the temperature, are plotted in Figure 13. The thermal sensitivities of the two DETFs are −27.6 ppm/°C and −28.8 ppm/°C, respectively. The temperature-induced stress is believed to be substantially relieved by the designed anchors with the isolation frame, since the TCFs of the DETF resonators are all around −28 ppm/°C and a good linear relationship is found between the frequency and the temperature, as analyzed in Section 2.3. The raw bias output of the SRA is shown in Figure 14a. The variation of the bias is about 70 mg over the temperature of the 100 °C range. The thermal sensitivity of the SRA is thus calculated to be 0.7 mg/°C, which is equivalent to about 0.16 Hz/°C. The thermal bias stability (1σ) of the SRA was approximately 1 mg after a third-order temperature compensation was applied, as displayed in Figure 14b, which is fairly good for a microelectromechanical systems (MEMS) accelerometer with an ±80 g measurement range.

The thermal sensitivity of the bias output is determined by temperature variations of the two DETF resonators. Ideally, the thermal drift of the bias can be cancelled by the differential readouts of the two DETF resonators. However, the structures of the two DETFs are not identical as expected, due to the fabrication error, which leads to the bias drift. Therefore, the initial frequency split of the two DETFs can be used as an indicator of the structural asymmetry that can affect the temperature characteristics of the SRA. Table 3 summarizes the measured performance of the proposed accelerometer and the comparison with the previously reported resonant accelerometers.

## 4. Conclusions

The SRAs received broad attention by the technical communities for their advantages of lower cost, a more miniature size, and the potential of navigation-grade performance. However, it is a challenging task to promote the temperature performance, since the SRAs are so sensitive to the temperature-induced stress. This paper proposed a SRA with stress insensitive anchor with an isolation frame that prevents the resonant beams from suffering the thermal stress along the sense axis, thus improving the bias stability. Besides, a high mechanical sensitivity was obtained by integrating the vibrating beams with the comb fingers without conventional additional mass design. The experimental results indicated that the nominal resonant frequency of the SRA was around 93 k Hz with the sensitivity and nonlinearity of 223.7 Hz/g and 5.1‰. The thermal sensitivities of the two resonant beams are −27.6 ppm/°C and −28.8 ppm/°C, respectively, which are very close to the theoretical value without the thermal stress effect. The bias thermal sensitivity and the stability (1σ) after compensation were tested to be approximately 0.7 mg/°C and 1 mg over the temperature range from −40 °C to 60 °C with an ±80 g measurement range. Future work will focus on the initial frequency reduction of the two DETF resonators to further improve the temperature stability of the SRA.

## Figures and Tables

**Table 1:** Structure parameters of the DETF.

Parameters	Value
Beam length of DETF (*L*)	600 μm
Beam width of DETF (*w*)	5.5 μm
Thickness of the structure (*h*)	60 μm
Finger width	5 μm
Finger length	20 μm
Finger gap	2.5 μm
Number of fingers	52

**Table 2:** Modal analysis results of the designed DETF.

Modal Order	Modal Shape	Resonant Frequency (Hz)
1st	translation mode	92905
2nd	drive mode	93065
3rd	in-phase wriggle mode	255960
4th	opposite wriggle mode	258590
5th	torsional mode	492900

**Table 3:** Summary of comparison with previous works.

Reference	[19]	[20]	[21]	[22]	[23]	This Work
Material	Silicon	Silicon	Silicon	Silicon	Silicon	Silicon
Frequency (Hz)	27934	3625	803950	25600	31462	93228
SF (Hz/g)	82	5.09	427	10	66.2	223.7
Freq. TCF (ppm/°C)	—	−28.9	−23	−29	58	−28.8
Bias TCF (mg/°C)	3.1	2.64	0.14	0.11	1.1	0.7
Temp. range (°C)	3–30	−40–40	−20–80	5–95	−40–60	−40–60
